# Regulation of Colonic Inflammation and Macrophage Homeostasis of IFN-γ-Primed Canine AMSCs in Experimental Colitis in Mice

**DOI:** 10.3390/ani14223283

**Published:** 2024-11-14

**Authors:** Chan-Hee Jo, Sang-Yun Lee, Young-Bum Son, Won-Jae Lee, Yong-Ho Choe, Hyeon-Jeong Lee, Seong-Ju Oh, Tae-Seok Kim, Chae-Yeon Hong, Sung-Lim Lee, Gyu-Jin Rho

**Affiliations:** 1 College of Veterinary Medicine, Gyeongsang National University, Jinju 52828, Republic of Korea; ch_jo@gnu.ac.kr (C.-H.J.); sy_lee@gnu.ac.kr (S.-Y.L.); yhchoego@gmail.com (Y.-H.C.); feellove97@naver.com (H.-J.L.); osj414@gnu.ac.kr (S.-J.O.); kiminfaith@naver.com (T.-S.K.); xoojoox8@gmail.com (C.-Y.H.); 2 Department of Obstetrics, College of Veterinary Medicine, Chonnam National University, 300 Yonbongdong, Buk-gu, Gwangju 500-757, Republic of Korea; ybson@jnu.ac.kr; 3 College of Veterinary Medicine, Kyungpook National University, Daegu 41566, Republic of Korea; iamcyshd@knu.ac.kr; 4 Research Institute of Life Sciences, Gyeongsang National University, Jinju 52828, Republic of Korea

**Keywords:** canine, mesenchymal stem cells, IFN-γ, inflammatory bowel disease, immunomodulation

## Abstract

Mesenchymal stem cells (MSCs) have shown potential in treating immune-mediated diseases due to their immunomodulatory properties, which can be enhanced by priming with inflammatory cytokines like interferon-gamma (IFN-γ). This study evaluates the therapeutic effects of IFN-γ-primed canine adipose tissue-derived MSCs (AMSCs) in a mouse model of inflammatory bowel disease (IBD). Canine AMSCs were primed with 50 ng/mL recombinant canine IFN-γ for 48 h, and the effects were compared to those seen in naïve (unprimed) AMSCs. IBD was induced in mice using dextran sodium sulfate (DSS), and AMSCs were injected intraperitoneally on days 1 and 3. The mice treated with IFN-γ-primed AMSCs showed improved clinical outcomes, including a reduced disease activity index (DAI), less body weight loss, and longer colon length compared to the mice treated with naïve AMSCs. A histological analysis revealed less damage to the intestinal structures and reduced inflammatory cell infiltration. IFN-γ priming led to a shift in the immune cell balance in the gut, decreasing pro-inflammatory macrophages (Ly6C^hi^) and increasing anti-inflammatory macrophages (Ly6C^lo^/MHC-II^hi^). This was associated with the reduced expression of inflammatory cytokine genes (Il-1β, Il-6, and Il-18) and increased expression of the intestinal stem cell marker Lgr5. These findings suggest that IFN-γ-primed AMSCs offer enhanced therapeutic potential for treating CE in veterinary medicine.

## Simple Summary

Mesenchymal stem cells (MSCs) can regulate the immune system, making them promising treatments for immune-related diseases in animals. Priming these cells with inflammatory signals, like interferon-gamma (IFN-γ), may enhance their effectiveness. This study explores the use of IFN-γ-primed adipose tissue-derived MSCs (AMSCs) from dogs in a mouse model of inflammatory bowel disease (IBD). The researchers compared the therapeutic effects of naïve and primed AMSCs. IBD mice treated with IFN-γ-primed AMSCs showed better health outcomes, including reduced disease severity, less weight loss, and longer colon length. A histological analysis revealed less damage to the intestinal structures and fewer inflammatory cells in the mice receiving the primed AMSCs. Further investigation showed that IFN-γ priming shifted the balance of immune cells in the gut, reducing harmful pro-inflammatory macrophages and increasing protective anti-inflammatory ones. This led to decreased inflammation and the increased expression of stem cell markers, promoting tissue repair. Overall, the IFN-γ-primed AMSCs showed a superior therapeutic potential in reducing inflammation and supporting gut healing. This suggests that primed AMSCs could be a promising new treatment for chronic enteropathy (CE) in veterinary medicine.

## 1. Introduction

Inflammatory bowel disease (IBD) refers to chronic inflammatory enteropathies characterized by uncontrolled inflammation in the gastrointestinal tract, affecting both humans and dogs, and leading to chronic diarrhea, vomiting, and weight loss [1,2,3]. Although the precise etiology of IBD remains elusive, it is influenced by multiple factors including environmental triggers, genetic factors, imbalance in the microbiota, and immune dysfunction [4]. In humans, IBD is classified into ulcerative colitis and Crohn’s disease [3]. In contrast, in veterinary medicine, the term chronic enteropathy (CE) is preferred to reflect the distinct clinical signs and treatment strategies in dogs and cats [5]. Clinically, CE is further classified based on the treatment response into antibiotic-responsive enteropathy (ARE), food-responsive enteropathy (FRE), immunosuppressant-responsive enteropathy (IRE), and non-responsive enteropathy (NRE) [5,6]. IRE, also known as steroid-responsive enteropathy, is considered the veterinary condition most similar to Crohn’s disease in humans [7]. 

Macrophages, as crucial components of the innate immune system, play several key roles in maintaining intestinal immune homeostasis [8,9,10]. The term “macrophage waterfall” refers to the sequential maturation process of monocytes into mature resident macrophages within the intestinal lamina propria [11]. In mice, the early stages of macrophage development are marked by high Ly6C expression, which decreases as maturation progresses. In the later stages, mature macrophages express the chemokine receptor CX3CR1 and upregulate MHC-II expression, indicating their transition to a resident macrophage state [11]. In a healthy gut microenvironment, resident macrophages contribute to gastrointestinal homeostasis by clearing pathogens and supporting the expansion of regulatory T cells in the lamina propria [12]. However, during inflammation, Ly6C^hi^ monocytes are recruited from the bloodstream into the colonic lamina propria, where they differentiate into an inflammatory macrophage phenotype [13,14]. These inflammatory macrophages produce mediators that accumulate in the mucosa, disrupting intestinal homeostasis and accelerating inflammation [13]. It is suggested that a decrease in resident macrophages, along with an increase in early differentiated macrophages, plays a key role in the pathogenesis of the disease, particularly in FRE and IRE [15]. Conventional immunosuppressive treatments such as glucocorticoids are associated with various adverse effects that limit their long-term application, emphasizing the need for alternative treatment strategies [5]. Mesenchymal stromal cells (MSCs) have emerged as a potential therapeutic option due to their ability to modulate intestinal inflammation by regulating inflammatory cytokines [16].

Mesenchymal stem cells (MSCs), known for their low immunogenicity, can modulate both the innate and adaptive immune systems, including T cells, macrophages, dendritic cells, and natural killer (NK) cells, through direct cell-to-cell contact and the secretion of paracrine factors [17,18]. Several studies have highlighted the interaction between MSCs and inflammatory macrophages, demonstrating that MSCs can induce a shift in the macrophage phenotype from the pro-inflammatory M1 phenotype to the anti-inflammatory M2 phenotype in vitro [19,20,21]. Given the significant role of macrophages in CE, stem cell-based therapies have emerged as promising therapeutic approaches, particularly due to their immunomodulatory properties [22,23,24]. 

MSCs exert their immunomodulatory ability in response to the host microenvironment; the pre-treatment of MSCs with pro-inflammatory cytokines to mimic the inflammatory milieu in advance can boost their anti-inflammatory effect [25,26,27]. Priming MSCs with pro-inflammatory factors, such as interferon-gamma (IFN-γ), tumor necrosis factor-alpha (TNF-α), interleukin 1 beta (IL-1β), and Interleukin-17 (IL-17), before delivery has been considered a promising strategy to enhance their therapeutic effects [28,29]. Among these cytokines, IFN-γ has been recognized as the most potent stimulator of key immunoregulatory factors, such as indoleamine 2,3-dioxygenase (IDO), prostaglandin E2 (PGE2), and hepatocyte growth factor (HGF) [30,31]. IFN-γ-primed MSCs showed an enhanced ability to suppress T cell proliferation via both direct and indirect contact, which was specifically related to increased IDO-1 and PGE2 concentrations [28,32]. Consequently, IFN-γ-primed MSCs have been employed as therapeutic agents in modulating T cell-related immune responses in various disease models, including experimental autoimmune encephalomyelitis (EAE) [33], renal fibrosis [34], and atopic dermatitis [35]. However, while the effects of IFN-γ-primed MSCs on T cell suppression have been extensively studied, their influence on macrophage polarization remains insufficiently explored and requires further investigation to establish their potential for immunosuppressive therapy in CE.

In this study, we first explored the modulatory effects of IFN-γ-primed canine adipose tissue-derived MSCs on macrophages in a DSS-colitis mouse model. We specifically evaluated the therapeutic potential of these primed MSCs by examining histological changes in intestinal structure, inflammatory cell infiltration, and, most notably, their impact on macrophage homeostasis within the lamina propria, providing new insights into their immunomodulatory functions.

## 2. Materials and Methods

### 2.1. Induction of IFN-γ Priming of Canine AMSCs

Canine AMSCs (cAMSCs) were isolated from the abdominal adipose tissue of clinically healthy beagles (n = 3). All procedures were approved by the Animal Center for Biomedical Experimentation at Gyeongsang National University (No. GNU-210329-M0033). The tissues were minced and digested with 0.1% collagenase type I in PBS at 37 °C for 1 h, followed by neutralization with Advanced DMEM (Dulbecco’s Modified Eagle Medium, ADMEM) containing 10% FBS and antibiotics. The cell suspension was filtered and centrifuged, and the pellets were resuspended in ADMEM supplemented with 10% FBS and antibiotics. Cells were cultured at 37 °C in a 5% CO_2_ incubator, with unattached cells removed by medium changes. Attached cells were harvested at 80–90% confluence and subcultured until passage 3–4. For priming, cAMSCs were seeded in 6-well plates and cultured in ADMEM with 10% FBS. After 12 h, the medium was replaced with ADMEM containing 50 ng/mL recombinant canine IFN-γ for 48 h at 37 °C in 5% CO_2_.

### 2.2. Establishment of DSS-Induced Colitis Mouse Model and Assessment of Colitis Disease Severity

To induce the acute colitis models, 8-week-old male C57BL/6 mice were administered 2.5% DSS (MP Biomedicals, Solon, OH) in drinking water for 7 days. All procedures were approved by the Animal Center for Biomedical Experimentation at Gyeongsang National University (No. GNU-210329-M003). The mice were randomly divided into the following four groups (n = 6 mice/group): (i) normal, (ii) DSS-only (colitis-induced mice injected with PBS), (iii) DSS-naïve cAMSCs (colitis-induced mice injected with non-primed AMSCs), (iv) DSS_IFN-γ_cAMSCs (colitis-induced mice injected with IFN-γ-primed cAMSCs). The mice were injected intraperitoneally (i.p.) with 3 × 10^6^ naïve or IFN-γ-primed cAMSCs diluted in 200 µL PBS on days 1 and 3, or the DSS-only group was injected with the same volume of PBS. All mice were sacrificed on day 10. In the mice with DSS-induced colitis injected with naïve or IFN-γ-activated cAMSCs, the severity of colitis was assessed daily by scoring the disease activity index (DAI), which includes evaluating stool consistency, body weight loss, and the presence of fecal blood. Body weight loss was scored as follows: score 0 (no body weight loss), score 1 (body weight loss within 1–5%), score 2 (body weight loss within 5–10%), score 3 (body weight loss within 10–20%), and score 4 (greater than 20% body weight loss). Stool consistency was determined as follows: score 0 (solid pellets), score 1 (soft but adherent in pellet shape), score 2 (loose stool but with some solidity), score 3 (loose stool with signs of liquid consistency), and score 4 (diarrhea). Blood presence in stool was determined as follows: score 0 (no blood), score 2 (red feces), and score 4 (visible bleeding). The score of each parameter was added to yield the final score.

### 2.3. Histological Analysis of Colon Tissues

The colon tissues of DSS-induced colitis mice were fixed in 4% formaldehyde, embedded in paraffin, and sectioned at 5 μm thickness. To analyze the pathological morphology of the colon, the sections were stained with hematoxylin and eosin (H&E). The severity of the disease was assessed by evaluating 5 factors: infiltration of inflammatory cells (0, none; 1, mild; 2, moderate; 3, severe), inflammation extent (0, none; 1, mucosa; 2, mucosa and submucosa; 3, transmural), crypt damage (0, none; 1, ~10% loss of crypt; 2, ~20% loss of crypt; 3, over 20% loss of crypt), loss of an epithelial cell (0, none; 1, ~5% loss of epithelial cell; 2, ~10% loss of epithelial cell; 3, over 10% loss of epithelial cell), and depletion of goblet cells (0, none; 1, mild; 2, moderate; 3, severe). The total histological score was calculated as the sum of each individual parameters, as explained in the Supplemental Table S1 and Supplemental Figure S1.

### 2.4. Isolation of Mesenteric Lymph Nodes (MLNs) and Colonic Lamina Propria Cells

Isolation of MLNs and colonic lamina propria cells from DSS-induced colitis mice was performed as previously described [36]. In brief, colons were carefully flushed of their luminal content with PBS to remove the feces. The colons were then opened longitudinally and cut into small pieces, and washed with PBS. To remove the colonic epithelium and mucus, colon tissues were incubated with PBS (without Ca^2+^ and Mg^2+^) containing 5% FBS, 2 mM EDTA, and 1 mM DTT for 30 min, at 37 °C. Subsequently, colon pieces were resuspended in the digestion media containing RPMI 1640 containing 5% FBS, 200 U/mL Collagenase VIII, and 150 μg/mL DNase I for 50 min at 37 °C, shaking at 250 rpm. After digestion, cell suspensions were filtered sequentially through 100 μm and 40 μm and cell strainer. Collected cell suspensions were then resuspended with RPMI containing 40% Percoll solution, overlaid with 80% Percoll gradient, and centrifuged at 900× *g* for 20 min. The isolated cells collected from the interface of the 40% and 80% Percoll gradient were washed with RPMI three times and used for further flow cytometry analysis.

### 2.5. Intracellular Cytokine Staining

Cells were obtained from the spleen using mechanical disruption. The isolated cells were stimulated with 40 ng/mL PMA (phorbol 12-myristate 13-acetate) and 1 μg/mL ionomycin in the presence of protein transport inhibitor (BD GolgiPlug, BD bioscience, San Diego, CA, USA) for 4 hrs at 37 °C. After stimulation, the cells were harvested and stained with fixable viability dye and PE-Cy7-conjugated anti-CD4 (clone GK1.5) antibody. Following surface antigen staining, fixation and permeabilization were performed by using intracellular fixation and permeabilization buffer set (eBioscience, San Diego, CA, USA) for staining intracellular antigens. After permeabilization, cytokine transcription factors were stained with PE-conjugated anti IL-4 (clone 11B11) and APC-conjugated anti-IL-17A (clone eBio17B7). The samples were analyzed by BD LSRFortessa™ X-20 Cell Analyzer (BD, Franklin lakes, NJ, USA).

### 2.6. Flow Cytometry Analysis

To evaluate the monocyte to macrophage phenotype in the colonic lamina propria of DSS-induced colitis mice, isolated cells were incubated with Fixable Viability Dye eFlourTM 506 for 30 min at 4 °C and followed by staining with surface antibodies; CD45 (clone 30-F11), CD11c (clone HL3), CD11b (clone M1/70), CD64 (clone X54-5/7.1), Ly-6C (clone AL-21), CX3CR1 (clone SA011F11), and MHC-Ⅱ (clone M5/114.15.2) for 30 min at 4 °C. Cells were then resuspended in FACS buffer and analyzed by BD LSRFortessa™ X-20 Cell Analyzer (BD, Franklin lakes, NJ, USA). All antibodies were purchased from Invitrogen (Waltham, WA, USA) and all data were analyzed using FlowJo v10 software.

### 2.7. RNA Extraction, cDNA Synthesis, and qRT-PCR Analysis

Total RNA was isolated using easy-spinTM Total RNA Extraction Kit (iNtRON Biotechnology, Seongnam, Republic of Korea) and quantified with a Nanodrop (Optizen NanoQ). A total of 500 ng RNA was synthesized into complementary DNA (cDNA) using HiSenScript^TM^ RH (-) RT PreMix Kits (iNtRON Biotechnology, Seongnam, Republic of Korea). The qRT-PCR reaction was performed using Rotor Gene Q (Qiagen, Hilden, Germany) with 50 ng cDNA quantified with RealMODTM Green AP 5x qPCR mix (iNtRON Biotechnology, Seongnam, Republic of Korea) supplemented with 10 μM specific primers. The PCR reaction cycle was as follows: initial activation at 95 °C for 12 min, followed by 40 cycles of PCR at 95 °C for 15 s, 60 °C for 25 s, and 72 °C for 25 s. Melting and amplification curves and cycle threshold values (Ct values) were then determined by Rotor-Gene Q series software (Qiagen). The primers used in this study are listed in Table 1.

### 2.8. Immunohistochemistry and Immunofluorescence Analysis

Paraffin-embedded colon tissue sections (5 μm) were deparaffinized in xylene baths and rehydrated sequentially in graded series (100%, 95%, 80%, and 70%) of ethanol solutions. The rehydrated colon sections were boiled in 0.01 M sodium citrate buffer for 20 min to retrieve the antigen and allowed to cool at room temperature. For IHC analysis, the sections were then incubated with 3% H_2_O_2_ for 30 min at room temperature to inhibit endogenous peroxidase activity, followed by incubation with 10% normal goat serum in PBS for 1 h at room temperature to block the non-specific antibody binding sites. The sections were consecutively treated with primary antibodies of F4/80 Rabbit mAb (Abcam, 1:100, Cat#: Ab111101) at 4 °C overnight, followed by washing with PBS and reacting with biotinylated goat anti-rabbit IgG secondary antibody (1:200) for 1 h at room temperature. After a subsequent washing with PBS, avidin-biotin-peroxidase complex (1:50, Vectastain Elite ABC kit) was applied for 30 min followed by peroxidase detection with a mixture of 3,3′-diaminobenzidine (DAB) to detect positive signals. All slides were counterstained with Mayer’s hematoxylin. For immunofluorescence analysis, after antigen retrieval using 0.01 M sodium citrate buffer for 20 min, the sections were then permeabilized with 0.2% Triton-X for 10 min and blocked with 10% normal goat serum for 1 h at room temperature. The sectioned slides were then incubated with a primary antibody of Lgr5 (Invitrogen, 1:100, Cat#: MA5-25644) at 4 °C overnight. After washing with PBS for three times, the sections were incubated with a secondary antibody, AlexaFlour 488-conjugated goat anti-mouse IgG (1:200), at room temperature for 1 h in darkness. The nuclei were stained with 1 μg/mL 4′,6-diamidino-2-phenylindole (DAPI) for 5 min and mounted in a Vectashield mounting medium (Vector Laboratories, NJ, USA). Immunohistochemical sections were observed at 200× magnification using a DM 4000 microscope (Leica, Germany) and analyzed using the Image J 1.44 software. After color filtering, DAB-marked areas were quantified and expressed as percent of total area. The fluorescence images were acquired using confocal microscopy (Olympus FV 1000) and analyzed using the Image J 1.44 software. To quantitate the number of Lgr5-positive cells per crypt, the total cell count was determined by identifying nuclei stained with DAPI. Lgr5-positive cells, displaying high fluorescence intensity, were counted as positive. All assessments were performed on at least 5 sections in each group under the blinded condition.

### 2.9. Statistical Analysis

All experimental data were analyzed using GraphPad Prism version 8. The statistical differences were analyzed by *t*-test and one-way analysis of variance (ANOVA) followed by Tukey’s multiple comparisons test. The data are presented as mean ± standard error of the mean (SEM), and statistical significance was defined as *p* < 0.05.

## 3. Results

### 3.1. Canine IFN-γ-Primed AMSCs Alleviate DSS-Induced Acute Colitis in Mice

**Figure 1:**
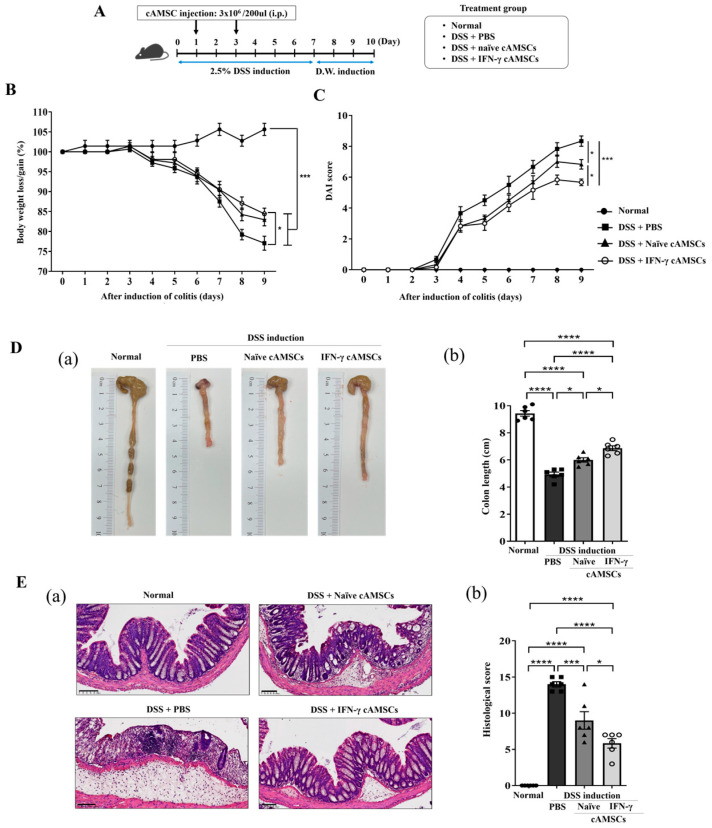
Therapeutic effects of IFN-γ-primed cAMSCs in a DSS-induced colitis model. (**A**) Colitis was induced by administering 2.5% DSS in drinking water for 7 days. Naïve and IFN-γ-primed cAMSCs were i.p. injected (3 × 10^6^ cells) into DSS-treated mice on days 1 and 3. The same volume of PBS was injected into DSS-treated mice as a positive control. (**B**) Body weight loss and (**C**) disease activity index (DAI) were monitored daily. (**D**,**E**) Colon length (b) and H&E staining (a) and histopathological score were analyzed. Scale bar = 100 µm. The filled circles, squares, triangles, and open circles represent data from normal mice, DSS treated mice with PBS, DSS treated mice with Naïve cAMSCs, and DSS treated mice with IFN-γ-primed cAMSCs, respectively. Data are represented as the mean ± SEM (n = 6 mice per group). * = *p* < 0.05, *** = *p* < 0.001, and **** = *p* < 0.0001.

To assess the immunomodulatory effects of the IFN-γ-primed cAMSCs, acute colitis was induced in mice using 2.5% DSS in drinking water over 7 days. The mice treated with the IFN-γ-primed cAMSCs exhibited significantly less body weight loss and a marked reduction in Disease Activity Index (DAI) scores compared to those receiving naïve cAMSCs (*p* < 0.05, Figure 1B,C). Furthermore, the colon length was better preserved in mice treated with naïve cAMSCs, with a significant improvement observed in those treated with IFN-γ-primed cAMSCs (*p* < 0.05 and *p* < 0.001, respectively; Figure 1D (a) and (b)). 

The histopathological analysis revealed that the mice with DSS-induced colitis displayed severe intestinal damage, characterized by the disruption of the intestinal architecture, transmural inflammatory cell infiltration, and the loss of goblet cells (Figure 1E). These pathological changes were significantly ameliorated following treatment with naïve cAMSCs and were further improved with IFN-γ-primed cAMSCs, as evidenced by a reduction in histological scores compared to the DSS-only controls.

### 3.2. Suppression of Pathogenic T Cells by Canine IFN-γ-Primed AMSCs

**Figure 2:**
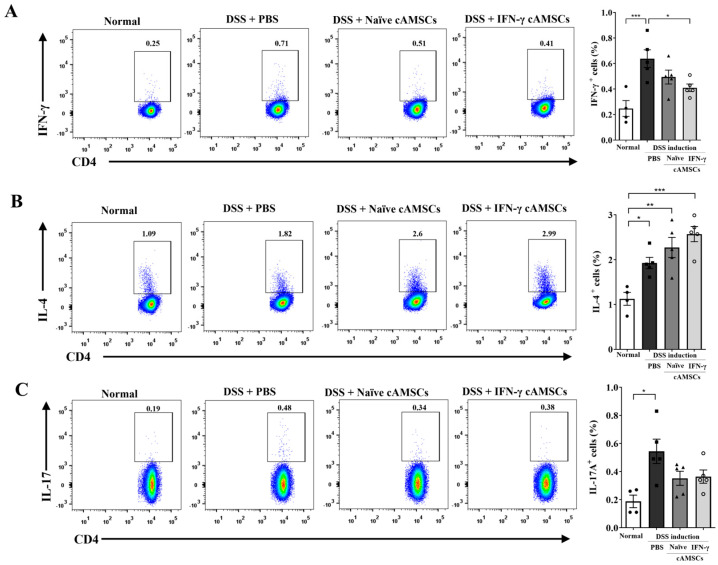
Inhibitory effect of IFN-γ-primed cAMSCs on T cell differentiation in mesenteric lymph nodes. Flow cytometry analysis of Th1, Th2, and Th17 cells in mesenteric lymph nodes (MLNs) of normal mice (n = 4), DSS−induced colitis mice (n = 5), DSS + naïve cAMSC−treated mice (n = 5), and DSS + IFN-γ-primed cAMSC−treated mice (n = 5). Representative plots showing the subpopulations of T cells: (**A**) IFN-γ-producing (Th1) cells; (**B**) IL-4-producing (Th2) cells; (**C**) IL-17-producing (Th17) cells. The filled circles, squares, triangles, and open circles represent data from normal mice, DSS treated mice with PBS, DSS treated mice with Naïve cAMSCs, and DSS treated mice with IFN-γ-primed cAMSCs, respectively. Data are represented as the mean ± SEM. * = *p* < 0.05, ** = *p* < 0.01, and *** = *p* < 0.001.

To explore the effects of IFN-γ-primed cAMSCs on Th cell differentiation, mesenteric lymph node (MLN) cells were isolated and analyzed by flow cytometry. The results showed increased populations of CD4+ IFN-γ+ Th1 cells, CD4+ IL-4+ Th2 cells, and CD4+ IL-17+ Th17 cells in the DSS-induced colitis mice (Figure 2). The administration of IFN-γ-primed cAMSCs significantly reduced the populations of Th1 cells when compared to the DSS-only controls. In addition, CD4+ IL-17+ Th17 cells tended to decrease in the mice with IFN-γ-primed cAMSCs compared to the DSS-induced colitis mice. In contrast, the percentage of Th2 cells showed a tendency to increase in the mice receiving naïve cAMSCs and IFN-γ-primed cAMSCs compared to the DSS-only controls.

### 3.3. Reduction in Ly6C^high^ Monocyte Accumulation in the Colonic Lamina Propria by Canine IFN-γ-Primed AMSCs

**Figure 3:**
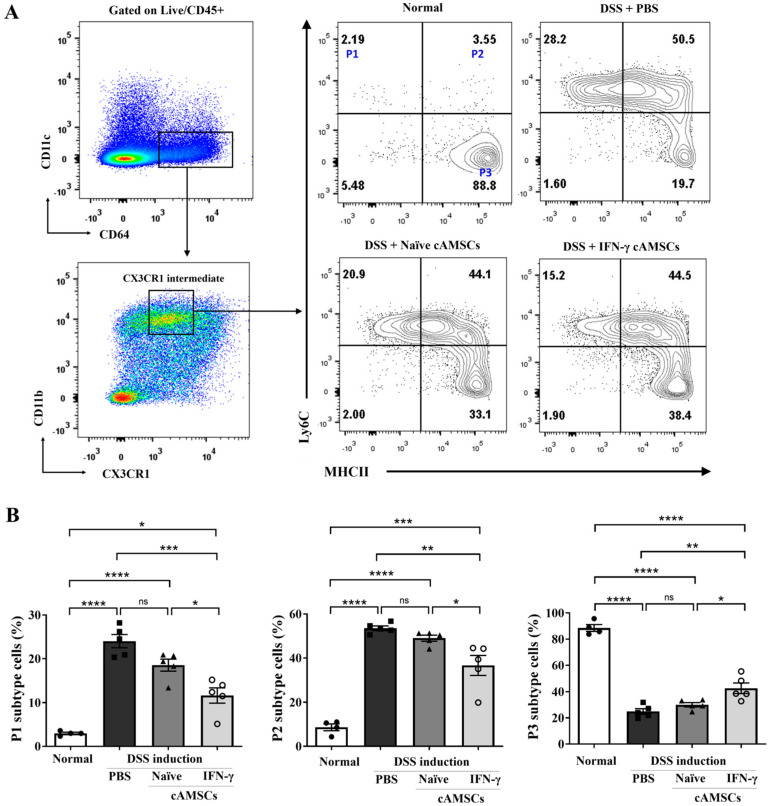
Inhibition of accumulation of Ly6C ^high^ macrophages in lamina propria by administering IFN-γ primed cAMSCs. (**A**) Representative plots showing the subpopulations of P1 (Ly6C ^hi^/MHCII ^lo^), P2 (Ly6C^hi^/MHC-Ⅱ int/^hi^), and P3 (Ly6C^lo^/MHC-Ⅱ^hi^) lamina propria macrophages as determined by flow cytometry. (**B**) The percentage of macrophage subpopulations in the lamina propria of normal mice (n = 4), DSS-induced colitis mice (n = 5), DSS + naïve cAMSC−treated mice (n = 5), and DSS + IFN-γ-primed cAMSC−treated mice (n = 5), as determined by flow cytometry. The filled circles, squares, triangles, and open circles represent data from normal mice, DSS treated mice with PBS, DSS treated mice with Naïve cAMSCs, and DSS treated mice with IFN-γ-primed cAMSCs, respectively. Data are represented as the mean ± SEM. ns = no significance, * = *p* < 0.05, ** = *p* < 0.01, *** = *p* < 0.001, and **** = *p* < 0.0001.

To characterize the state transition from monocyte to macrophage, cells from colonic lamina propria were categorized into three distinct populations based on the expression levels of Ly6C and MHC-Ⅱ: P1 (Ly6C^hi^/MHC-Ⅱ^lo^), P2 (Ly6C^hi^/MHC-Ⅱint/^hi^), and P3 (Ly6C^lo^/MHC-Ⅱ^hi^). Population 1 (P1) cells expressed a high level of Ly6C, indicating “pro-inflammatory” monocytes derived from blood. Population 2 (P2) cells expressed a high level of Ly6C and intermediate to high expression of MHC-Ⅱ, indicating a transition phase from monocytes to macrophages. Population 3 (P3) cells expressed a high level of MHC-Ⅱ and lacked Ly6C, indicating “anti-inflammatory” macrophages (Figure 3A). Compared to the normal mice, the infiltration of blood-derived monocytes expressing lymphocyte antigen 6C-high (Ly6C^hi^) was increased in the DSS-induced colitis mice. Interestingly, these pro-inflammatory P1 and P2 (Ly6C^hi^) cells were decreased in the mice treated with naïve cAMSCs and further with the IFN-γ-primed cAMSCs. Furthermore, anti-inflammatory P3 macrophages were significantly (*p* < 0.05) increased in the mice treated with IFN-γ-primed cAMSCs compared to the mice treated with naïve cAMSCs (Figure 3A,B).

### 3.4. Reduction in Inflammatory Cytokines and Macrophage Infiltration in the Colon by Canine IFN-γ-Primed AMSCs

**Figure 4:**
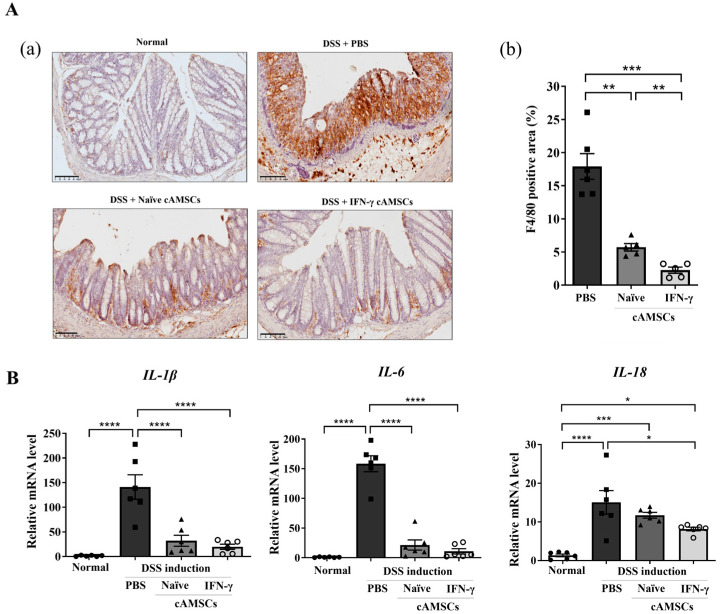
Effects of IFN-γ-primed cAMSCs on macrophage infiltration and intestinal stem cell regeneration. (**A**) F4/80-positive macrophage infiltration was analyzed by immunohistochemistry (a) and quantified per slide section using ImageJ software (b). (**B**) The mRNA expression of inflammatory cytokines (IL-1β, IL-6, and IL-18) secreted by macrophages in colon tissue was analyzed. The filled circles, squares, triangles, and open circles represent data from normal mice, DSS treated mice with PBS, DSS treated mice with Naïve cAMSCs, and DSS treated mice with IFN-γ-primed cAMSCs, respectively. Data are represented as the mean ± SEM. * = *p* < 0.05, ** = *p* < 0.01, *** = *p* < 0.001, and **** = *p* < 0.0001.

In the DSS-induced colitis mice, the number of F4/80+ macrophages in the colon was significantly (*p* < 0.01) reduced by administering IFN-γ-primed cAMSCs compared to with the naïve canine ASMCs (Figure 4A). The increased number of macrophages also correlated with elevated mRNA expression levels of pro-inflammatory cytokines, including Il-1β, Il-6, and Il-18 in the DSS-induced colon tissue. The treatment with naïve cAMSCs significantly (*p* < 0.0001) reduced these mRNA expression levels, and this reduction was further enhanced with the treatment with IFN-γ-primed cAMSCs (Figure 4B). Overall, the administration of IFN-γ-primed cAMSCs effectively down-regulated pathogenic macrophage accumulation, leading to a corresponding reduction in the gene expression levels of inflammation-related cytokines.

### 3.5. Induction of Regenerative Ability in Colonic Epithelial Cells by IFN-γ-Primed cAMSCs

**Figure 5:**
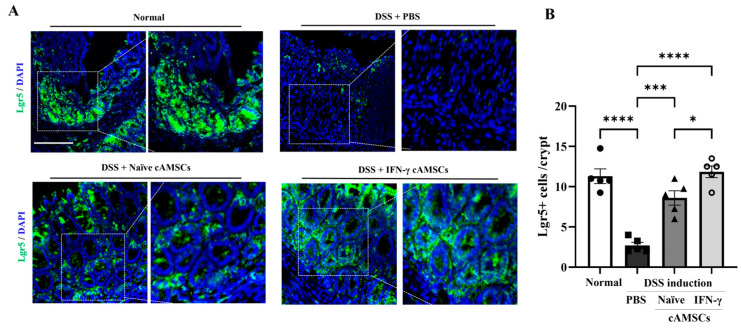
Effects of IFN-γ-primed cAMSCs in the regeneration of intestinal stem cells. (**A**) Representative images of immunofluorescence staining for Lgr5 in colon tissue from normal mice, DSS-colitis mice, DSS + naïve cAMSC-treated mice, and DSS + IFN-γ-primed cAMSC-treated mice (green: Lgr5, blue: DAPI). Scale bar =100 μm. (**B**) The quantification of Lgr5-positive cells per crypt (n = 5). The filled circles, squares, triangles, and open circles represent data from normal mice, DSS treated mice with PBS, DSS treated mice with Naïve cAMSCs, and DSS treated mice with IFN-γ-primed cAMSCs, respectively. Data are represented as the mean ± SEM. * = *p* < 0.05, *** = *p* < 0.001, and **** = *p* < 0.0001.

The ability to maintain intestinal stem cells was evaluated by analyzing the expression of Lgr5-positive cells through immunofluorescence analysis. Lgr5, a marker of intestinal stem cells, was significantly (*p* < 0.05 and *p* < 0.01, respectively) increased in the mice treated with both IFN-γ-primed and naïve cAMSCs compared to in the DSS-induced colitis mice (Figure 5). 

## 4. Discussion

MSCs have emerged as a promising therapeutic approach for CE due to their immunomodulatory and regenerative properties [22,23]. In particular, recent studies have focused on enhancing the immunomodulatory potential of MSCs through priming strategies in cellular therapy [25,37]. Specifically, priming MSCs with the inflammatory cytokine IFN-γ has been shown to enhance their immunosuppressive effects on T lymphocytes and monocyte/macrophages [32,38]. Previous studies have demonstrated that IFN-γ-primed MSCs show enhanced immune-modulatory abilities in various immune-mediated diseases including EAE [33], atopic dermatitis [35], and multiple sclerosis [39]. In this regard, several experimental studies utilizing human MSCs primed with IFN-γ have been conducted for the treatment of IBD, demonstrating their efficacy and safety for therapeutic application [40,41,42]. However, despite the growing prevalence of CE in dogs [6], the application of stem cell therapy using primed canine MSCs remains underexplored. Therefore, this study aims to evaluate the therapeutic effects of canine AMSCs primed with IFN-γ in a DSS-colitis mouse model. Additionally, we focused on examining the monocyte-to- macrophage maturation process (macrophage waterfall) in mice following treatment with IFN-γ-primed cAMSCs.

Our results showed that the intraperitoneally injected IFN-γ-primed cAMSCs significantly improved the therapeutic efficacy more than injecting naïve cAMSCs in a DSS-induced colitis model. The body weight loss and DAI score were further improved in the mice treated with IFN-γ-primed cAMSCs compared to with the naïve cAMSCs. Moreover, the colon length and histological analysis further demonstrated the enhanced therapeutic effects of the IFN-γ-primed cAMSCs. Previous studies have revealed that canine MSCs primed with pro-inflammatory cytokines, such as a combination of IFN-γ and TNF-α [43] or TNF-α alone [44], can significantly enhance their immunomodulatory effects in colitis models. 

Macrophages are key immune cells of the innate immune system that play a crucial role in maintaining intestinal homeostasis [14]. Given their important role, several studies have focused on the immunomodulatory effects of MSCs on promoting M2 macrophage polarization [21,43,44]. Previous research has shown that TNF-α-primed canine AMSCs enhance immunomodulatory effects in a DSS-colitis model by increasing M2 macrophages in the colon [44]. In line with these findings, our study further explored the monocyte-to-macrophage maturation process in colonic lamina propria. During intestinal inflammation, Ly6C^hi^ monocytes are predominantly recruited into the colonic lamina propria and terminal differentiation to mature resident macrophages is disrupted. This leads to the accumulation of pro-inflammatory Ly6C^hi^ macrophages in inflamed tissue [45]. Therefore, selectively targeting Ly6C^hi^ monocytes/macrophages can be a promising therapeutic strategy for regulating the immune response. Furthermore, another study demonstrated that the immunoregulatory effect of BM-MSCs in reducing hepatic fibrosis is facilitated by the conversion of Ly6C^hi^ macrophages into Ly6C^lo^ macrophages [46]. Our results showed a significant (*p* < 0.001) reduction in pro-inflammatory macrophages, specifically the P1 (Ly6C^hi^/MHCII^lo^) and P2 (Ly6C^hi^/MHCIIinter/^hi^) subtypes, following administration with IFN-γ-primed cAMSCs. Additionally, anti-inflammatory macrophages, identified as the P3 (Ly6C^lo^/MHCII**^hi^**) subtype, were significantly (*p* < 0.05) increased in mice treated with IFN-γ-primed cAMSCs compared to those treated with naïve cAMSCs. This shift in macrophage populations was associated with a downregulation in the expression of pro-inflammatory cytokine genes, including Il-1β, Il-6, and Il-18, in colon tissue. These findings provide novel insights into the immunomodulatory role of IFN-γ-primed canine AMSCs in regulating Ly6C^hi^ monocyte/macrophage accumulation in the colonic mucosa.

Our findings showed that the IFN-γ-primed cAMSCs reduced the Th1 population while enhancing Th2 cells, with no significant change in the Th17 population in the colitis mice. Consistent with our findings, previous studies have [47] shown that MSCs downregulate Th1 and Th17 responses while upregulating Th2- and Treg-mediated responses, contributing to the improvement in colonic inflammation [47,48]. Moreover, Th2 cells play a crucial role in maintaining the integrity of the intestinal mucosal barrier. Upon epithelial cell damage, Th2 cells are activated, leading to the secretion of Th2-related cytokines that promote tissue repair [49,50].

Moreover, treatment with IFN-γ-primed cAMSCs not only decreased inflammation in the colon tissue but also significantly increased the intestinal stem cell populations. The observed decrease in F4/80+ macrophage infiltration in the colonic mucosa and increased Lgr5+ intestinal stem cell populations further support the role of IFN-γ-primed cAMSCs in preserving colonic epithelial integrity and alleviating inflammation. Lgr5+ stem cells are critical factors for maintaining cellular turnover and tissue homeostasis; however, colitis induces a loss of these cells, leading to the disruption of crypt structure [51]. A previous study showed that MSCs also stimulated significant proliferation of intestinal epithelial cells, leading to increased numbers of Lgr5+ intestinal stem cells [52]. Similarly, our results also demonstrated that the DSS treatment resulted in the depletion of Lgr5+ intestinal stem cells in the colon, but their expression was restored after administering the IFN-γ-primed cAMSCs. While these findings are encouraging, it remains unclear whether the observed increase in the Lgr5+ stem cells directly correlates with the complete functional recovery of the epithelial barrier. In this study, we primarily focused on the initial phases of regeneration, and as such, the long-term sustainability of this increase in Lgr5+ stem cells was not assessed. It is possible that, although Lgr5+ cells were restored, their persistence and the full restoration of epithelial barrier function may require longer-term analysis. Future investigations should therefore explore whether the effects of IFN-γ-primed cAMSCs on Lgr5+ stem cells are sustained over time and whether this leads to the comprehensive recovery of the epithelial barrier, including tests of tissue integrity and functionality.

Our study suggest that IFN-γ-primed cAMSCs could preserve Lgr5+ intestinal stem cells and may play a significant role in the regeneration of damaged colonic tissue. Therefore, this study demonstrated the enhanced therapeutic efficacy of IFN-γ-primed cAMSCs in a DSS-induced colitis model by regulating the accumulation of Ly6C^hi^ monocyte/macrophages in the colonic lamina propria and promoting regenerative effects in intestinal stem cells. 

## 5. Conclusions

In conclusion, this study demonstrates that IFN-γ-primed cAMSCs significantly downregulated the accumulation of Ly6C^hi^ monocyte/macrophages in the colonic lamina propria and promoted the regeneration of intestinal stem cells in a DSS-colitis mouse model. These results underscore the potential of IFN-γ-primed AMSCs for improving treatment outcomes in experimental colitis. Further investigation into the underlying mechanisms of these effects is crucial for advancing MSC-based therapies for immune-mediated diseases in canines.

## Figures and Tables

**Table 1:** qRT-PCR primer sequences of inflammation-related genes.

Target Gene	Sequence	Product Size (bp)	Anneal. Tm. (°C)	Accession
*Il-1β*	F: ATGACCTGTTCTTTGAAGTTGACG R: CCTGAAGCTCTTGTTGATGTGC	128	60	BC011437.1
*Il-6*	F: GGCCTTCCCTACTTCACAAG R: ATTTCCACGATTTCCCAGAG	126	60	NM_001314054.1
*Il-18*	F: GACTCTTGCGTCAACTTCAAGG R: TTTGTCAACGAAGAGAACTTGG	159	60	NM_008360.2
*Tbp*	F: AGTGAAGAACAATCCAGACTAG R: TATAGGGAACTTCACATCACA	129	60	NM_013684.3

## Data Availability

The original contributions presented in the study are included in the article, further inquiries can be directed to the corresponding author.

## Supplementary Materials

The following supporting information can be downloaded at https://www.mdpi.com/article/10.3390/ani14223283/s1: Table S1: Histological scoring system for DSS-induced colitis; Figure S1: Representative images corresponding to histological scoring criteria.
