# Supplementary material for: Regulation of Colonic Inflammation and Macrophage Homeostasis of IFN-γ-Primed Canine AMSCs in Experimental Colitis in Mice
Source: Animals (Basel). 2024 Nov 14;14(22):3283. doi: 10.3390/ani14223283 (PMC11591378; doi:10.3390/ani14223283)
Supplement: Supplementary file 1 [file animals-14-03283-s001.zip › animals-3238368-supplementary.pdf]

animals

MDPI

Supplementary Materials

Table S1. Histological scoring system for DSS-induced colitis.

Histological Scoring System		
**Infiltration of inflammatory cells**	0	None
	1	Mild
	2	Moderate
	3	Severe
**Inflammation extent**	0	None
	1	Mucosa
	2	Mucosa & Submucosa
	3	Transmural
**Loss of epithelium**	0	None
	1	Mild (~5% loss)
	2	Moderate (~10% loss)
	3	Severe (>10% loss)
**Crypt damage**	0	None
	1	Mild (~10% loss)
	2	Moderate (~20% loss)
	3	Severe (>20% loss)
**Depletion of goblet cells**	0	None
	1	Mild
	2	Moderate
	3	Severe

Supplementary Figure S1. Representative Images Corresponding to Histological Scoring Criteria

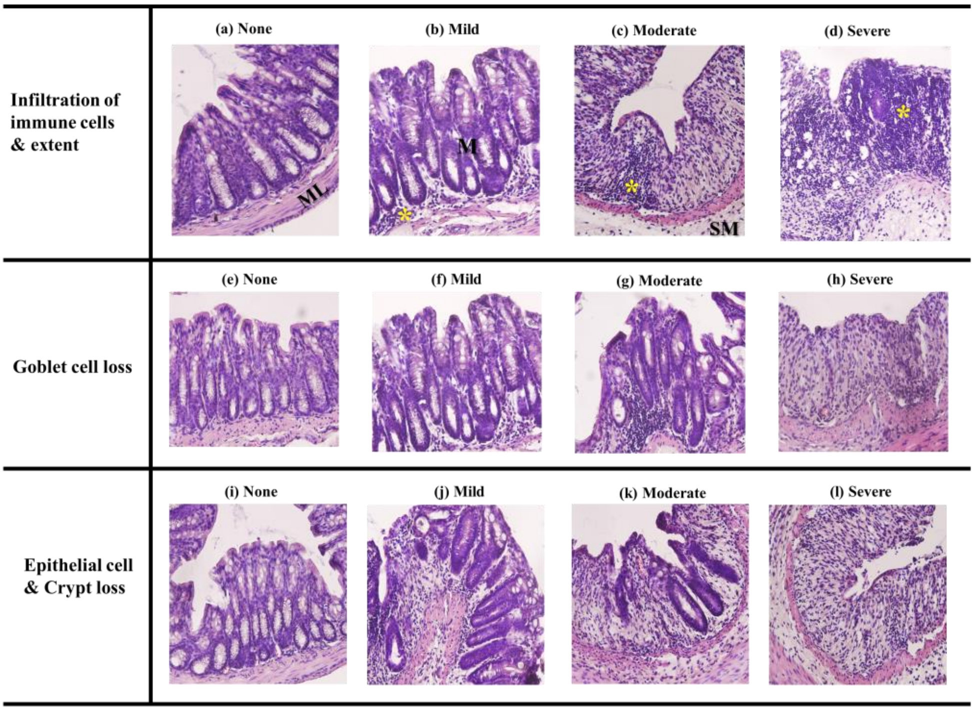

Figure S1. The severity of the disease was assessed by evaluating (**a**–**d**); infiltration of immune cells and extent, (**e**–**h**); goblet cell loss, and (**i**–**l**); epithelial cell and crypt loss. Yellow asterisks reflect the immune cells infiltration. M, mucosa; SM, submucosa; ML, muscular layer.
